# Magnetoelectric Tuning of Pinning‐Type Permanent Magnets through Atomic‐Scale Engineering of Grain Boundaries

**DOI:** 10.1002/adma.202006853

**Published:** 2020-12-23

**Authors:** Xinglong Ye, Fengkai Yan, Lukas Schäfer, Di Wang, Holger Geßwein, Wu Wang, Mohammed Reda Chellali, Leigh T. Stephenson, Konstantin Skokov, Oliver Gutfleisch, Dierk Raabe, Horst Hahn, Baptiste Gault, Robert Kruk

**Affiliations:** ^1^ Institute of Nanotechnology Karlsruhe Institute of Technology (KIT) 76344 Eggenstein‐Leopoldshafen Germany; ^2^ Department of Microstructure Physics and Alloy Design Max‐Planck‐Institut für Eisenforschung GmbH (MPIE) 40237 Düsseldorf Germany; ^3^ Department of Material Science Technical University Darmstadt 64287 Darmstadt Germany; ^4^ Karlsruhe Nano Micro Facility Karlsruhe Institute of Technology (KIT) 76131 Karlsruhe Germany; ^5^ Institute for Applied Materials Karlsruhe Institute of Technology 76344 Eggenstein‐Leopoldshafen Germany; ^6^ Department of Materials Imperial College London London SW7 2AZ UK; ^7^ Present address: Department of Physics Southern University of Science and Technology Shenzhen China; ^8^ Present address: Institute of Metal Research Chinese Academy of Sciences Shenyang China

**Keywords:** grain boundaries, hydrogen, magnetoelectric coupling, permanent magnets

## Abstract

Pinning‐type magnets with high coercivity at high temperatures are at the core of thriving clean‐energy technologies. Among these, Sm_2_Co_17_‐based magnets are excellent candidates owing to their high‐temperature stability. However, despite intensive efforts to optimize the intragranular microstructure, the coercivity currently only reaches 20–30% of the theoretical limits. Here, the roles of the grain‐interior nanostructure and the grain boundaries in controlling coercivity are disentangled by an emerging magnetoelectric approach. Through hydrogen charging/discharging by applying voltages of only ≈1 V, the coercivity is reversibly tuned by an unprecedented value of ≈1.3 T. In situ magneto‐structural characterization and atomic‐scale tracking of hydrogen atoms reveal that the segregation of hydrogen atoms at the grain boundaries, rather than the change of the crystal structure, dominates the reversible and substantial change of coercivity. Hydrogen reduces the local magnetocrystalline anisotropy and facilitates the magnetization reversal starting from the grain boundaries. This study opens a way to achieve the giant magnetoelectric effect in permanent magnets by engineering grain boundaries with hydrogen atoms. Furthermore, it reveals the so far neglected critical role of grain boundaries in the conventional magnetization‐switching paradigm of pinning‐type magnets, suggesting a critical reconsideration of engineering strategies to overcome the coercivity limits.

## Graphical abstract

Through injection of hydrogen atoms into grain boundaries, the coercivity of a Sm_2_Co_17_‐based magnet is tuned by ≈1.3 T by applying ≈1 V. This work not only offers an effective route to achieve giant magnetoelectric effects in metallic ferromagnets, but also unveils the neglected critical role of grain boundaries in determining the coercivity of pinning‐type magnets.

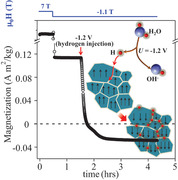

Permanent magnets with the ability to maintain their magnetization, i.e., a property referred to as coercivity, at high temperatures are crucial materials for serving the rapidly growing clean energy technologies, such as the electric vehicle and wind power.^[^ ^1^, ^2^, ^3^ ^]^ Improving high‐temperature magnetic properties of the currently used NdFeB and SmCo_5_ magnets is, however, challenging. To further raise the operating temperature, pinning‐type magnets, in which the coercivity arises from the pinning of magnetic domain walls at nanoprecipitates within the grains, are the most attractive candidates.^[^ ^4^, ^5^, ^6^ ^]^ For instance, Sm_2_Co_17_‐based magnet is the only candidate for use in electric motors working above 300 °C owing to its high Curie temperature and excellent temperature stability.^[^ ^7^, ^8^, ^9^, ^10^, ^11^ ^]^ Its coercivity is usually believed to be controlled exclusively by domain‐wall pinning because of the nanoscale cellular microstructure within the grain, while the initial demagnetization at grain boundaries is considered irrelevant. However, despite decades‐long efforts to optimize its intragranular microstructure, the coercivity currently only reaches 20–30% of the theoretical anisotropy field.^[^ ^1^, ^12^, ^13^ ^]^ Hence, it inevitably opens the question about the possible influence of grain boundaries during the magnetization reversal of the material.

The role of grain boundaries in pinning‐type magnets may be understood if they can be modified separately from the grain interior, in conjunction with measuring the associated coercivity change. Traditional processing approaches, such as heat treatments,^[^ ^11^ ^]^ plastic deformation,^[^ ^14^ ^]^ and alloying,^[^ ^7^, ^15^ ^]^ can dramatically change the coercivity. However, these approaches often induce irreversible modification (or destruction) of the microstructure both in grain boundaries and grain interior, obscuring the assessment of their respective impact on coercivity. Consequently, decoupling the separate roles of the grain boundary and the grain interior in magnetization reversal is technically challenging.

It has recently been demonstrated that the magnetoelectric approach can reversibly modify the magnetic properties of materials by applying external voltages without changing their microstructure.^[^ ^16^, ^17^, ^18^, ^19^, ^20^ ^]^ In particular, the voltage‐controlled hydrogen insertion/extraction into the crystal structure can substantially tune the coercivity of bulk ferromagnetic metallic materials such as the single‐crystalline SmCo_5_.^[^ ^21^ ^]^ Under voltage control, hydrogen atoms diffuse into the interstitial sites and modify the magnetocrystalline anisotropy drastically, which induces a change in coercivity. It is expected that in polycrystalline magnets, owing to the different trapping energy and mobility of hydrogen atoms to the different types of microstructural defects, hydrogen atoms would diffuse first along the grain boundaries, and then into the grain interiors.^[^ ^22^, ^23^ ^]^ This microstructural diffusion pattern, if mapped and understood with the aid of atomic‐scale observation of the location of the hydrogen atoms, offers an opportunity to decouple the roles of the grain boundary and the grain interior in polycrystalline pinning‐type magnets when the associated coercivity change is monitored at each step. Here, by employing electrochemically controlled hydrogen charging/discharging,^[^ ^24^ ^]^ we tuned the coercivity of the Sm_2_Co_17_‐based hard magnet by ≈1.3 T, the largest values ever achieved by magnetoelectric approaches.^[^ ^16^, ^17^, ^18^, ^19^, ^20^, ^21^ ^]^ The combined in‐ situ magneto‐structural measurements and atomic‐scale mapping of the hydrogen distribution^[^ ^25^, ^26^, ^27^ ^]^ reveal that hydrogen atoms strongly segregate at grain boundaries, which weakens the local magnetic anisotropy and accounts for the predominant change in coercivity. Our study opens a way to achieve giant magnetoelectric effects through atomic‐scale engineering of grain boundaries, and unveils the critical role of grain boundaries in limiting the performance of pinning‐type magnets, a mechanism that can be exploited for future optimization strategies.

The commercial Sm_2_Co_17_‐based permanent magnets were used with the compositions of Sm(Co_0.766_ Fe_0.116_Cu_0.088_ Zr_0.029_)_7.35_ (hereafter referred to as SmCo_7.35_ sample) (Table S1, Supporting Information). Its hysteresis loop shows an initial coercivity of ≈2.8 T (**Figure** **1**A). By magneto‐optical Kerr effect (MOKE) microscopy, its magnetization reversal process was observed under the demagnetization fields. Prior to MOKE imaging, the sample was fully magnetized at –6.8 T (Figure 1B), and the domain structure imaged with the *c*‐axis in the viewing plane.

**Figure 1:**
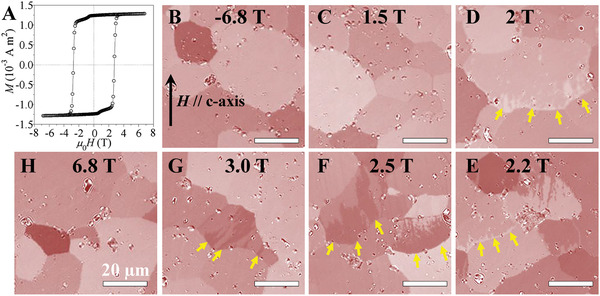
MOKE microscopy observation of the magnetization reversal process in Sm_2_Co_17_‐based magnet. A) Hysteresis loops of the bulk Sm_2_Co_17_‐type magnet. B–H) MOKE observation of the magnetic domain structure under different demagnetization field from the magnetically saturated state—the applied magnetic field is parallel to *c*‐axis of crystal structure as indicated in (B).

At a demagnetization field of 1.5 T, the sample retained its fully magnetized state (Figure 1C). When the field increased to 2 T, the reversed domains started to appear at grain boundaries (Figure 1D), and at 2.2 T, they expanded into the interior of the grains (Figure 1E). With further increasing field, the magnetic domains moved massively into the grain (Figure 1F). At 3.0 T, only a few residual domains were unreversed (Figure 1G). These observations demonstrate the initial nucleation of magnetic domains at grain boundaries before their growth into the grain interior.

An electrochemical three‐electrode cell was employed to charge/discharge the SmCo_7.35_ sample with hydrogen atoms (Figure S1, Supporting Information). In this setup, the as‐prepared electrode with the SmCo_7.35_ particles was the working electrode and 1 m KOH aqueous solution the electrolyte (Figure S2, Supporting Information). During hydrogen charging, the electrochemical reduction of water molecules on the metal surface provides the hydrogen adatoms that subsequently diffuse into the material. Conversely, during the discharging, the hydrogen atoms on the surface (H_ads_) are oxidized and removed, resulting in hydrogen desorption. According to the measured cyclic voltammogram (Figure S3, Supporting Information), the voltages steps of –1.2 and –0.4 V were used to charge and discharge the sample, respectively. –0.4 V was chosen because it exceeds the potential for reversible hydrogen adsorption/desorption (–1.01 V) and was neither too high nor positive (when above 0 V), which could trigger oxygen evolution and oxidation of the material.

The response of the coercivity of the as‐prepared SmCo_7.35_ sample to hydrogen charging/discharging was explored using an in situ superconducting quantum interference device (SQUID). The coercivity of the as‐prepared sample was ≈2.3 T (**Figure** **2**A), slightly lower than that of the bulk‐form pristine sample (≈2.8 T) (Figure 1A), possibly due to the damage and the misalignment of the particles during the preparation of the electrode.^[^ ^12^ ^]^ After charging at –1.2 V for 1 h, the coercivity drastically decreased to ≈1.0 T. We monitored the recovery of the coercivity during the discharging process by continuously recording the hysteresis loops. The coercivity increased monotonically with the discharging time (Figure 2A), and regained most of its initial value in the early stage of the discharging process, reaching ≈1.9 T within 10 h (inset in Figure 2A). After a prolonged time of discharging (≈55 h), the coercivity fully recovered. Yet, after charging and discharging the remanence remained slightly lower than that of the pristine sample and the second quadrant demagnetization curve exhibited a soft shoulder, which may be ascribed to the irreversible oxidation at the surface.

**Figure 2:**
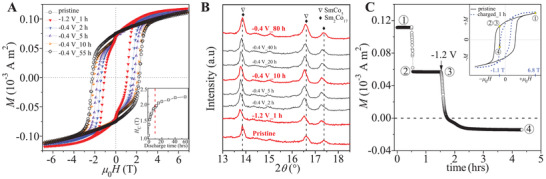
Reversible modification of the coercivity in Sm_2_Co_17_‐based magnet by voltage‐controlled hydrogen charging/discharging. A) Hysteresis loops of the as‐prepared sample and those after hydrogen charging at –1.2 V for 1 h (charged_1h) and after further discharging for 2 h (DC_2h), 5 h (DC_5 h), 10 h (DC_10 h), and 55 h (DC_55 h). Inset shows the continuous change of the coercivity against the discharging time. B) In situ XRD monitoring the evolution of the crystal structure of the as‐prepared sample during charging and discharging, showing that within the first 10 h of discharging, the diffraction pattern remained nearly unchanged compared with the charged sample. C) Time evolution of the magnetization in the as‐prepared sample with the voltages switched to –1.2 V, showing the immediate response of magnetization reversal to voltage stimulus. Points ①, ②, ③, and ④ indicate different magnetization states as shown in the inset.

In parallel, we studied the dynamics of the hydrogen charging/discharging process by observing the evolution of crystal structure with in situ X‐ray diffraction (XRD) in transmission mode (Figure 2B). Upon hydrogen‐charging, all diffraction peaks were shifted to lower angles, and after 1 h, the positions of the diffraction peaks stayed unchanged, indicating the complete charging of the whole sample. In the discharging process, two stages were discerned. First, strikingly, only a negligible shift of the peaks was observed over the first 10 h (Figure 2C), indicating that the bulk material is still charged with hydrogen atoms. Second, only after about 80 h of discharging, the peaks recovered to their original positions. This observation is in strong contrast with the substantial change in coercivity over the corresponding period (inset in Figure 2A). It suggests that the predominant coercivity change is not ascribed to the slow hydrogen desorption from the volume of the material.

Since the predominant change of coercivity does not arise from the volumetric slow diffusion of hydrogen atoms, we expected a relatively fast response of magnetization reversal to hydrogen charging (Figure 2C). The as‐prepared sample was first magnetized with a large magnetic field of 6.8 T (point ① in the inset). Then, the magnetic field was reversed to –1.1 T (point ②), smaller than the coercivity of the pristine sample (≈2.3 T), and therefore the magnetization remained positive and nearly constant. Upon hydrogen charging by applying –1.2 V (point ③), the magnetization decreased immediately and abruptly, and flipped from positive to negative in ≈10 min. The magnetization started to level off after ≈4 h. The immediate response of magnetization reversal to the voltage stimulus confirms the existence of relatively fast diffusion path of hydrogen atoms.

To rationalize the fast diffusion pathways of hydrogen atoms, multiscale multi‐microscopy mapping of the micro‐ and nanostructural features were carried out. Optical microscopy (Figures 1 and **3**A) shows that the sample was polycrystalline with grains of ≈26 µm separated by high‐angle grain boundaries (HAGBs). The grains were further divided into subgrains by low‐angle grain boundaries (LAGBs) as shown by electron back‐scattered imaging (Figure 3B). Inside the grain, transmission electron microscopy shows the typical cellular structure, composed of the matrix cell with sizes of ≈40 nm, the cell boundary and the Zr‐rich lamellae crossing the cellular structure (TEM, Figure 3C; Figure S4, Supporting Information). After tilting the *c*‐axis of the specimen out of the viewing plane, high‐resolution TEM and the corresponding selected‐area electron diffraction show that the matrix phase is Sm_2_Co_17_ (rhombohedral Th_2_Zn_17_ type) and the cell boundary phase SmCo_5_ (hexagonal CaCu_5_ type) (Figure 3D). This hierarchical micro‐ and nanostructure matches previous reports.^[^ ^7^, ^8^, ^9^, ^10^, ^11^ ^]^

**Figure 3:**
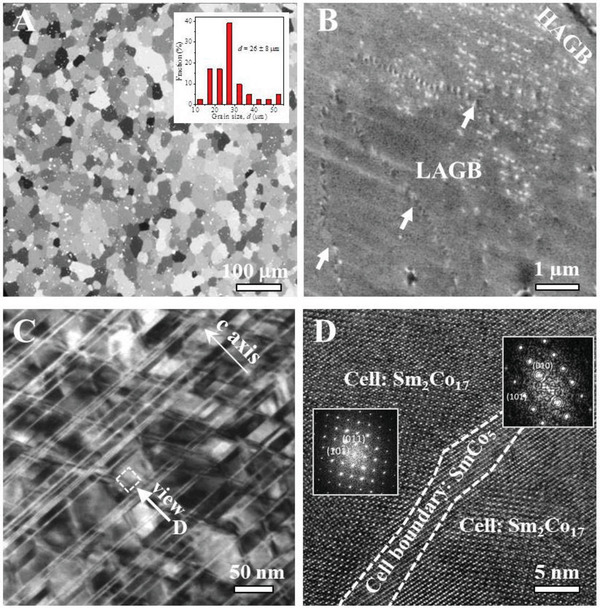
Multilevel multi‐spectroscopy characterization of the hierarchical microstructure in Sm_2_Co_17_‐based magnet. A) An optical microscopy image showing the polycrystalline grains with grain sizes ≈26 µm. B) An enlarged image of the crystal grain showing LAGBs within the grain. C) A bright‐field TEM image within the grain showing the nanoscaled cellular structure, composed of the Sm_2_Co_17_ cell, the SmCo_5_ cell boundary, and the Zr‐rich platelets. D) A close‐up on the cell and cell boundary by HRTEM and their Fourier transformation. The *c*‐axis of the matrix phase in (C) and (D) is in and out of the viewing plane, respectively.

Atom probe tomography (APT) was used to locate hydrogen atoms and deuterium atoms within the hierarchical microstructure of the deuterium‐charged samples. Isotopic marking by deuterium atoms (D) minimized the influence of residual hydrogen in the atom probe. We first analysed a specimen containing a HAGB (Figures S5 and S6, Supporting Information). The element‐specific atom maps in **Figure** **4**A reveal the Cu‐rich cell boundaries, the matrix cells, and the Zr‐rich platelets, matching TEM results. 3D reconstruction shows that D mostly segregates in a 10–12 nm thick layer at HAGB, reaching a concentration of ≈3.5 at% (Figure 4A). A close face‐on view (Figure 4B) reveals that D segregates at the intersection of the cell boundaries with the HAGB. In the cells and cell boundaries, a very limited amount of D was detected within the structure close to the detection limit, with no noticeable partitioning difference between them (Figure S6, Supporting Information). In addition, D atoms appear slightly depleted from the Zr‐rich phase.

**Figure 4:**
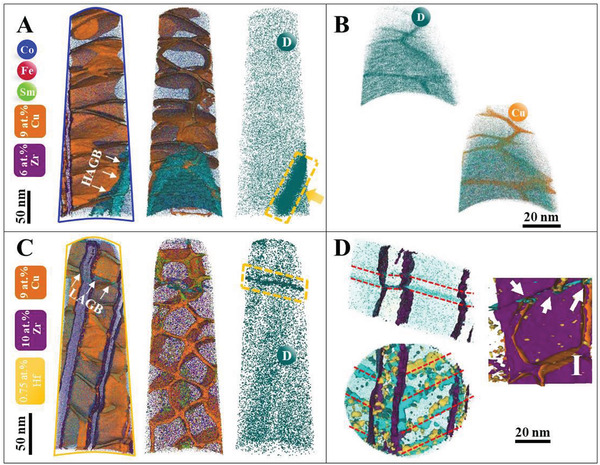
Atomic‐scale tracking of hydrogen/deuterium atoms within the microstructure in Sm_2_Co_17_‐based magnet. A) 3D atom map of a deuterium‐charged sample containing a HAGB. The sets of 9 at% Cu and 6 at% Zr iso‐composition surfaces evidence the Cu‐rich cell boundaries and the Zr‐rich phase; the distribution of deuterium reveals strong planar segregation near HAGB. B) A face‐on view on the distribution of D and Cu using 3.5 at% D and 9 at% Cu near the HAGB, highlighting the co‐location of D and Cu. Note the incomplete cellular structure and its abrupt stop at HAGB. C) 3D atom map of a deuterium‐charged specimen containing an LAGB. Note the disruption of the cellular structure adjacent to LAGB. The distribution of D shows the strong planar segregation at LAGB, the depletion at Zr‐rich phase, and no partioning between cell and cell boundary. D) A close‐up using 0.35 at% D, 0.75 at% Hf, and 4.5 at% H iso‐surfaces showing D segregation along a series of linear features interpreted as dislocations, each of which is connected to a cell edge; a close‐up on the LAGB showing the direct connection of each dislocation to a cell boundary as indicated by white arrows (right side in (D)).

Further analysis was performed to target LAGBs (Figure 4C,D; Figure S7, Supporting Information). Again, deuterium appears depleted in the Zr‐rich phase, and no preferential segregation within cells and the cell boundaries (Figure S8, Supporting Information). Yet again, a strong segregation of deuterium at LAGB was observed. The corresponding top view shows a series of linear features highlighted by a set of 0.35 at% D iso‐composition surfaces, which are likely the dislocations that constitute the LAGB.^[^ ^26^ ^]^ The D‐concentration at these dislocations can reach up to 0.4 at% D, and H up to 4–5 at%. Importantly, the cell edges are all connected to these dislocations and the cell structure stops abruptly at LAGB (Figure 4D).

The observation of hydrogen/deuterium segregation at GB regions (Figure 4), coupled with in situ XRD, detecting no volumetric structural change (Figure 2B), suggests that the substantial change of coercivity in the early stage of discharging process arises from the desorption of hydrogen atoms from GBs. The ability to modulate the coercivity by only charging/discharging GBs enables the fast control of coercivity, as verified by the immediate start of magnetization reversal upon hydrogen charging (Figure 2B). The identified critical role of grain boundaries in controlling the coercivity explains why reducing the volume fraction of grain boundaries^[^ ^28^ ^]^ or optimizing the cellular structure near the grain boundary^[^ ^29^ ^]^ can increase the coercivity of Sm_2_Co_17_‐based magnets. Below we discuss how hydrogen segregation at GBs changes the coercivity, starting with the microstructural features near GBs.

Three microstructural features distinguish the GB region from the grain interior. First, compared with the continuous cellular structure in the grain interior (bottom part in Figure 4C), the cell boundaries are broken and terminate at GBs (Figure 4A,C,D). Second, the typical cell size and shape in the grain is ≈40 nm and with a regular shape, but becomes larger and strongly elongated near GBs (Figure 4). These results agree with recent TEM reports that show that the incomplete cellular structure near GBs extend towards the sub‐micrometer scale.^[^ ^30^, ^31^ ^]^ Third, the composition profiles (Figures S6 and S8, Supporting Information) show that the SmCo_5_ phase contains almost twice as much Cu near the GB, i.e., 30 at% compared with 15 at% in the grain interior.

The observed different cellular structure and microchemistry near GBs significantly reduces the local nucleation field required for magnetization reversal. According to the micromagnetic theory, the critical nucleation field, *H_n_*, can be described by^[^ ^12^ ^]^

(1) $$\begin{array}{c} H_{n}=\frac{1}{2M_{s}\Delta }\left(\gamma_{{\text{SmCo}}_{5}}-\gamma_{\text{GB}}\right)-N_{d}M_{s} \end{array}$$

in which ∆ is the width of the transition region where the domain‐wall energy changes from γ_GB_ in the GBs to $\gamma_{{\text{SmCo}}_{5}}$ in the cell boundary, and *N*_d_ is the demagnetizing factor. The SmCo_5_ phase has much larger magnetocrystalline anisotropy than GBs, and determines the domain‐wall energy difference, $\left(\gamma_{{\text{SmCo}}_{5}}-\gamma_{\text{GB}}\right)$.Near GBs, the Cu concentration in the SmCo_5_ phase becomes twice that in the grain interior, which substantially reduces its magnetocrystalline anisotropy^[^ ^32^, ^33^ ^]^ and thus the domain wall energy, $\gamma_{{\text{SmCo}}_{5}}$. In addition, the disrupted SmCo_5_ phase allows the easy movement of domain walls through the matrix phase, triggering a macroscopic magnetization reversal. These account for the preferential nucleation of reversed domains near GBs (Figure 1). Moreover, when the SmCo_5_ phase was charged with hydrogen, its magnetocrystalline anisotropy was shown to decrease by ≈40%.^[^ ^21^ ^]^ This further decreases the domain‐wall energy of the SmCo_5_ phase and the nucleation field. Hydrogen segregation may also enlarge the transition region (Δ) between the GBs and the SmCo_5_ phase with its continuous concentration change and reduce the nucleation field. Hence, hydrogen segregation acts here as a tool to further weaken the nucleation field of the GB region and amplify its effect in initiating the magnetization reversal. Next, we consider the mechanism behind the propagation of the initially‐nucleated magnetic domains near GBs into the grain interior.

In the grain interior, the continuous network of the SmCo_5_ phase subdivides the individual grains of the Sm_2_Co_17_ matrix into a nanoscale cellular structure, rendering them into classical pinning‐type magnets. However, the models to explain their magnetization reversal assume that grain boundaries should be non‐ferromagnetic and one‐to‐two atomic layers thick to reduce the associated stray field negligibly.^[^ ^12^, ^34^ ^]^ This is not the case in the current material because of the expanded region near GBs with the disintegrated cellular structure and different microchemistry. We can describe the demagnetization process of the whole grain triggered by the initial demagnetization near GB as follows. The local magnetic field is a superposition of the external field *H*_ext_ and the local demagnetization field, *N*_d_ʹ*M*, where *N*_d_ʹ is the local or effective demagnetization factor and *M* is the net magnetization of the sample. The latter can be significantly inhomogeneous, and reach values much larger than the net demagnetization field,^[^ ^35^ ^]^ *H*_D_ = *N*_d_ *M*, *N*_d_ being the demagnetization factor of the sample. As discussed earlier, the nucleation field of the GB region, *H*_c,GB_, is much smaller than *H*_c_ of the grain interior, *H*_c,g_. Under a small external field, we have *H*_c,g_ > *H*_c,GB_ > *H*_ext_ + *N*_d_ *M*. As the local magnetic field, *H*_ext_+ *N*_d_ *M*, approaches *H*_c,GB_, the initial nucleation of magnetic domains occurs near GBs (Figure 1), producing a local demagnetization field, *N*_d_ʹ*M*_s_, where *M*_s_ is spontaneous magnetization of the main Sm_2_Co_17_ phase. Then, the adjacent inner layer with higher coercivity, *H*_c,g_, is under the higher magnetic field, *H*_ext _+ *N*_d_ *M *+ *N*_d_ʹ*M*_s_. This additional negative field, *N*_d_ʹ*M*_s_, can be of 0.5–1.0 T, depending on microstructural features and *M*_s_.^[^ ^35^ ^]^ Thus, if *H*_c,g_ – *H*_c,GB _< *N*_d_ʹ*M*_s_, the demagnetization of GB region will inevitably trigger an avalanche‐like demagnetization process in the whole grain, driven by the local enhancement of the demagnetization field.

Our results have shown that by electrochemically controlled hydrogen charging/discharging the coercivity of the pinning‐type Sm_2_Co_17_‐based magnet can be tuned by an unprecedented value of ≈1.3 T, the highest value ever reported by a magnetoelectric approach. In situ magneto‐structural characterization and atomic‐scale tracking of hydrogen atoms over the hierarchical microstructure reveals that the predominant change of the coercivity arises from the decoration of the grain boundaries with hydrogen atoms. The demonstrated voltage‐controlled engineering of grain boundaries with hydrogen opens up a way to achieve the reversible and giant modification of magnetic properties for various applications such as magnetoelectric actuation or sensing in which large magnetoelectric effects are needed.^[^ ^36^ ^]^ Our findings also reveal, in contrary to the conventional magnetization‐switching paradigm, a critical role of the grain boundaries in determining the coercivity, and are anticipated to apply to other technologically important pinning‐type magnets such as MnAl^[^ ^4^, ^5^ ^]^ and FePt.^[^ ^6^ ^]^ Future performance‐optimization strategies should be reconsidered to enhance the coercivity of pinning‐type magnets for the clean‐energy applications, such as via atomic‐scale engineering of grain boundaries.

## Experimental Section

### Materials and Microstructure Characterization

The Sm_2_Co_17_‐type permanent magnets with dimensions of Φ10 mm × 6 mm were purchased from Sigma‐Aldrich (stock no. 692832). Its composition was analyzed by inductively coupled plasma mass spectroscopy (Table S1, Supporting Information) and its microstructure characterized by optical microscopy (KIT), field‐emission scanning electron microscopy (SEM) equipped with energy‐dispersive X‐ray spectroscopy, and electron channeling contrast imaging (ECCI) (Zeiss Ultra 600/Merlin, both at KIT and MPIE), and transmission electron microscope (TEM, FEI Titan 80‐300, KIT). Before optical and SEM characterization, the sample surface was mechanically polished. The preparation of TEM samples followed the ordinary procedure of cutting, lifting, and milling using FIB/SEM dual beam system (FEI Strata 400 and Zeiss Auriga 60, KIT). The TEM observations were taken both with the *c*‐axis of the crystal structure out of and parallel with the viewing planes.

### MOKE Measurement

The magnetization reversal process was monitored by characterizing the magnetic domain structure under the external magnetic field using MOKE microscopy (Zeiss Axio Imager, D2m Evico Magnetics GmbH, TU Darmstadt). Before the MOKE observation, the sample was fully magnetized at a pulsed field of 6.8 T. Then, the magnetic domain structure was observed after applying a reversed magnetic field of 1.5, 2, 2.2, 2.5, 2.8, 3.0, and 6.8 T. The magnetic field was applied parallel to *c*‐axis of the matrix Sm_2_Co_17_ phase, and the images taken with the *c*‐axis in the viewing plane. To enhance the image contrast, the nonmagnetic background image was subtracted from the collected average image using KerrLab software.

### Preparation of the Sm_2_Co_17_ Electrode and the Electrochemical Setup

To prepare the Sm_2_Co_17_ electrode, the as‐received bulk sample was first charged with hydrogen atoms. The insertion of hydrogen atoms caused the expansion of the sample, and, consequently, the surface of the bulk sample collapsed into the very large particles. Then, the established procedure of Ye et al.^[^ ^20^ ^]^ was followed to prepare the powders into the Sm_2_Co_17_ electrode. Briefly, the particles were mixed with PVDF solution, coated onto thin copper foils under a homogeneous magnetic field to magnetically align the particles, and finally compressed under ≈100 MPa after drying. The charging and discharging were carried out under potentiostatic control in a three‐electrode electrochemical system (Autolab PGSTAT 302N, KIT). The working, the counter, and the reference electrodes were the Sm_2_Co_17_ electrode, Pt wires, and a pseudo‐Ag/AgCl electrode, respectively. As established by Ye et al.,^[^ ^21^ ^]^ the used electrolyte was an aqueous electrolyte of 1 m KOH. The potential of the pseudo‐Ag/AgCl electrode is 0.300 ± 0.002 V more positive than the standard Hg/HgO (1 m KOH) electrode, and for comparison, all the voltages in were converted to the Hg/HgO scale.

### In Situ XRD and SQUID Measurement

The crystal structure of the Sm_2_Co_17_ electrode under the application of –1.2 and –0.4 V was monitored by in situ XRD with a parallel beam laboratory rotating anode diffractometer (Mo Kα radiation) in transmission geometry. The herein electrochemical setup is the same as that established by Ye et al.,^[^ ^21^ ^]^ only that the working electrode is replaced by the Sm_2_Co_17_ electrode. Diffraction patterns were collected every 10 min with a Pilatus 300K‐W area detector. NIST SRM660b LaB_6_ powder was used for the detector calibration and the determination of the instrumental resolution. In situ magnetic measurement was carried out with a custom‐built miniaturized Teflon electrochemical cell in a SQUID (MPMS3, KIT) at room temperature. The in situ electrochemical cell inside SQUID and the measuring conditions are the same as that built up by Ye et al.,^[^ ^21^ ^]^ but the working electrode was replaced with the Sm_2_Co_17_ electrode.

### APT Measurement

For the APT (MPIE) measurement of hydrogen distribution, the Sm_2_Co_17_ electrode was charged at –1.2 V for ≈2.5 h in 0.1 m NaOD in D_2_O (instead of H_2_O) using the three‐electrode system as described above. After the full charging, the sample was cleaned by ethanol and was transferred in 10 min to FIB chamber for the cutting, milling, and lifting at room temperature (FEI Helios Nanolab 600/600i). Three to four APT tips were prepared within 3–4 h at room temperature. Annular milling was used to sharpen the needle‐shaped morphology with a diameter less than ≈100 nm. After that, a cleaning of the specimen at 5 kV was performed to remove the beam‐damaged surface regions. The prepared tips were transferred into the load‐lock chamber of APT, waited ≈2 h until the vacuum reached ≈10^−8^ Pa (LEAP 3000 XHR), and then transferred into analysis chamber (≈10^−11^ Pa) at 70 K. Data on Cameca LEAP 5000XR were also acquired, and only waited for half an hour before transferring the sample from APT load‐lock to analysis chamber. The APT experiments were conducted using high‐voltage mode with a pulse fraction of 15% at a base temperature of 70 K, a pulse frequency of 200 kHz, and an evaporation rate of 0.5. Atom probe data reconstruction and analysis were performed using CAMECA IVAS 3.8.4 software.

## Conflict of Interest

The authors declare no conflict of interest.

## Author Contributions

X.Y., R.K., and H.H. conceived the project. X.Y. designed the experiments. X.Y. conducted material preparation and microstructural observation, built in situ electrochemical charging/discharging setup, and conducted magnetic measurements. F.Y. conducted APT, part of SEM, assisted by L.T.S. F.Y. and B.G. processed the APT data. L.S. and X.Y. performed MOKE. H.G. and X.Y. performed in situ XRD. M.R.C. prepared TEM samples and discussed results. D.W. and W.W. conducted TEM observation. K.S. and O.G. contributed significantly to the interpretation of results. X.Y. wrote the initial draft. B.G., K.S., and R.K. revised the manuscript. All authors participated in the discussions, contributed to improving the manuscript, and approved the submitted manuscript.

## Supporting information

Supporting Information

## Data Availability

All data needed to evaluate the conclusions of the study are present in the article or the Supporting Information.
