# Supplementary material for: Magnetoelectric Tuning of Pinning‐Type Permanent Magnets through Atomic‐Scale Engineering of Grain Boundaries
Source: Adv Mater. 2020 Dec 23;33(5):2006853. doi: 10.1002/adma.202006853 (PMC11468041; doi:10.1002/adma.202006853)
Supplement: Supplementary file 1 — Supporting Information [file ADMA-33-2006853-s001.pdf]

© 2020 Wiley-VCH GmbH

ADVANCED MATERIALS

Supporting Information

for *Adv. Mater.*, DOI: 10.1002/adma.202006853

Magnetoelectric Tuning of Pinning-Type Permanent Magnets through Atomic-Scale Engineering of Grain Boundaries

Xinglong Ye, Fengkai Yan, Lukas Schäfer, Di Wang, Holger Geßwein, Wu Wang, Mohammed Reda Chellali, Leigh T. Stephenson, Konstantin Skokov, Oliver Gutfleisch, Dierk Raabe, Horst Hahn, Baptiste Gault,* and Robert Kruk*

Supporting information for

Magneto-electric Tuning of Pinning-Type Permanent Magnets through Atomic-Scale Engineering of Grain Boundaries

*Xinglong Ye**, *Fengkai Yan, Lukas Schäfer, Di Wang, Holger Geßwein, Wu Wang,*

*Mohammed Reda Chellali, Leigh T. Stephenson, Konstantin Skokov, Oliver Gutfleisch, Dierk Raabe, Horst Hahn, Baptiste Gault**, *Robert Kruk*

Dr. X. L. Ye, Dr. D. Wang, Dr. W. Wang♪, Dr. M. R. Chellali, Prof. H. Hahn, Dr. R. Kruk

Institute of Nanotechnology, Karlsruhe Institute of Technology (KIT), Eggenstein-Leopoldshafen, Germany

Email: xing-long.ye@kit.edu

Dr. F. K. Yan,♠ L. T. Stephenson, Prof. D. Raabe, Prof. B. Gault,

Department of Microstructure Physics and Alloy Design, Max-Planck-Institut für Eisenforschung GmbH (MPIE), Düsseldorf, Germany.

Email: b.gault@mpie.de

L. Schäfer, Dr. K. Skokov, Prof. O. Gutfleisch

Department of Material Science, Technical University Darmstadt, Darmstadt, Germany

Dr. D. Wang, Dr. W. Wang

Karlsruhe Nano Micro Facility, Karlsruhe Institute of Technology (KIT), Karlsruhe, Germany

Dr. H. Geßwein

[5] Institute for Applied Materials, Karlsruhe Institute of Technology, Eggenstein-Leopoldshafen, Germany.

Prof. B. Gault

Department of Materials, Imperial College London, London, UK

♠present address: Institute of Metal Research, Chinese Academy of Science, Shenyang, China

♪present address: Department of Physics, Southern University of Science and Technology, Shenzhen, China.

This PDF file includes:

Materials and methods

Fig. S1 to S8

Table S1

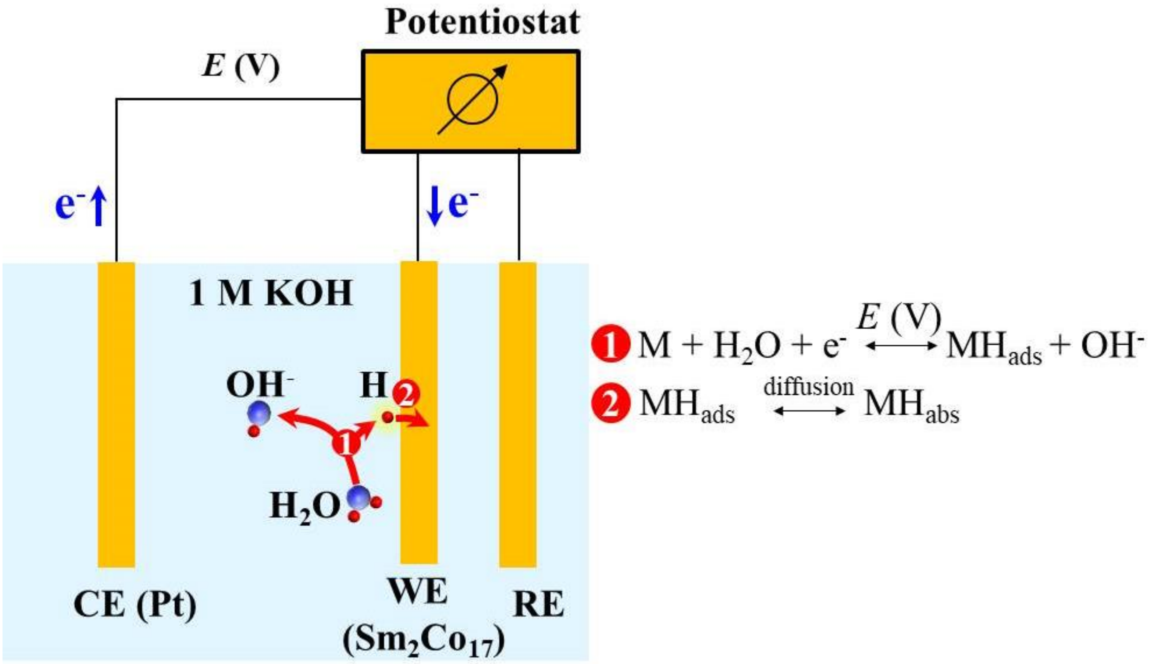

Fig. S1 Schematic of the voltage-controlled charging and discharging in Sm_2Co_{17}–based magnet with hydrogen atoms. WE, working electrode (Sm_2Co_{17}-based magnet); CE, counter electrode; RE, reference electrode (Hg/HgO electrode). Hydrogen atoms originate from the electrochemical reduction of water molecules at the metal (M)/electrolyte interface (reaction ①). They first adsorb onto the metal surface (H_{ads}), and then, driven by the concentration gradient, diffuse into the interstitial sites of the crystal structure (H_{abs}, reaction ②). For clarity, the discharging process is not depicted.

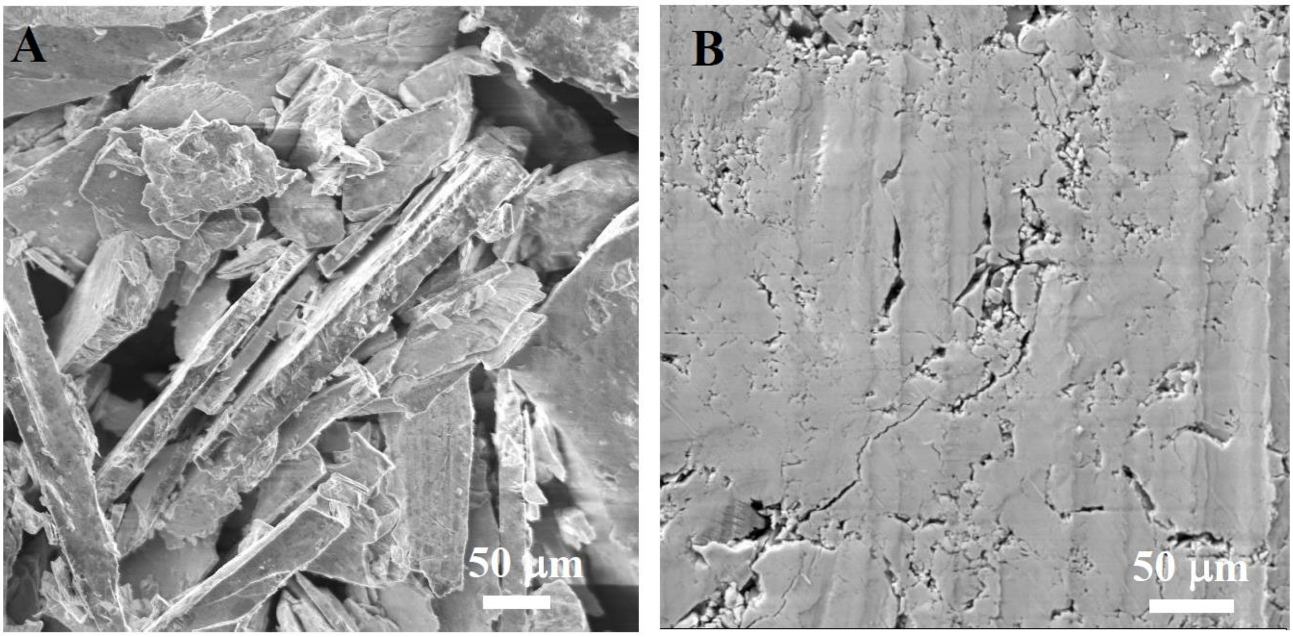

Fig. S2 An SEM image of the as-prepared powder (**A**) and the surface of the as-prepared $SmCo_{7.35}$ electrode (**B**).

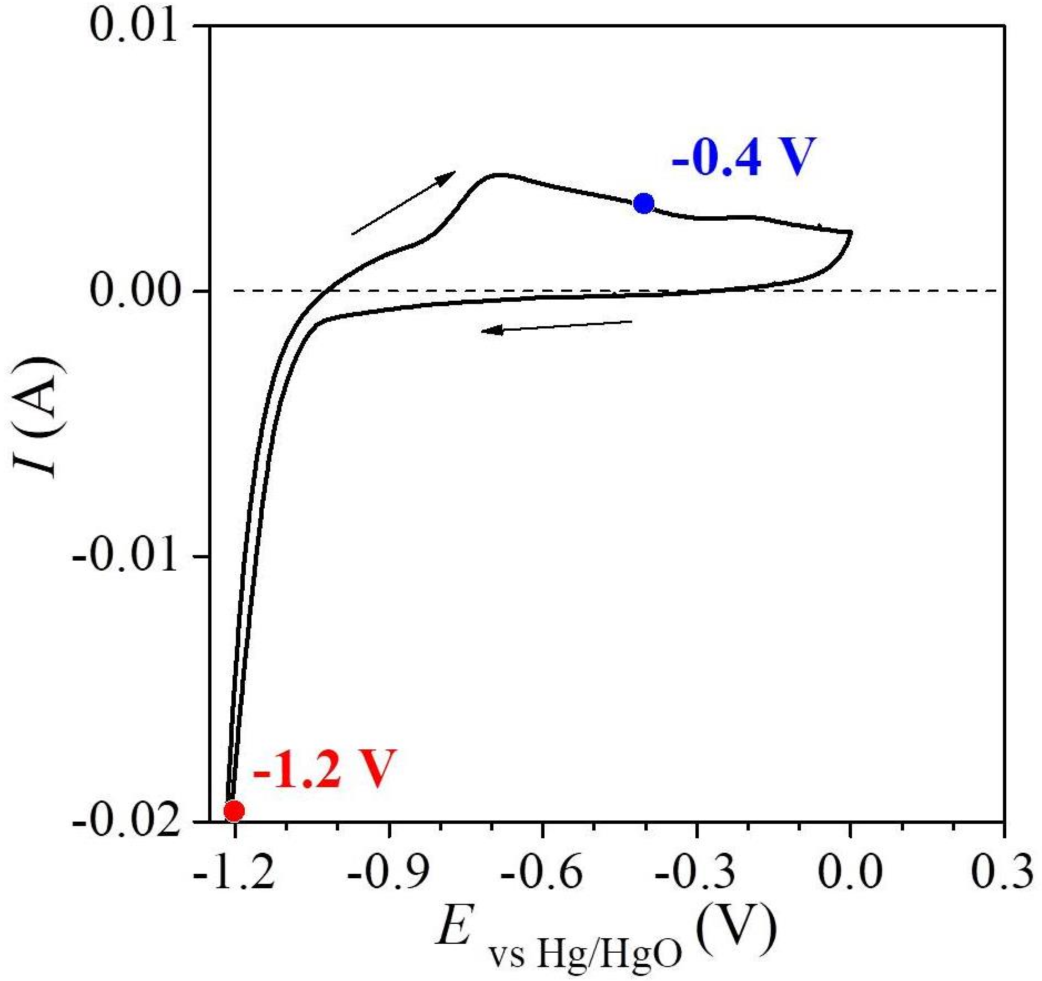

Fig. S3 A cyclic voltammogram curve of the Sm_2Co_{17} electrode in 1 M KOH with a scan rate of 2 mV s^{-1}. Based on this curve, the voltages of -1.2 V and -0.4 V were used to induce hydrogen charging and discharging, respectively. The voltage was with respect to Hg/HgO electrode.

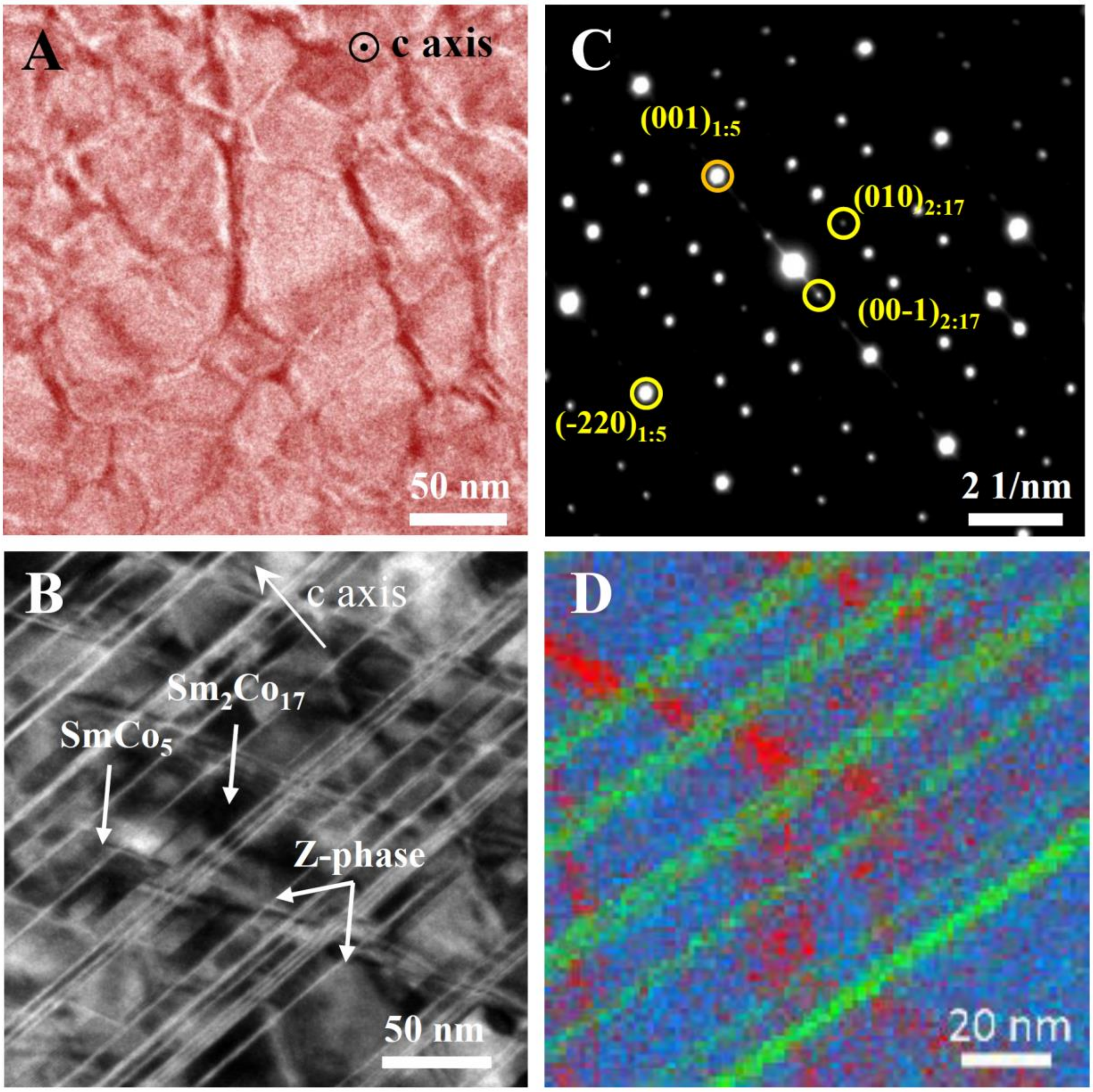

Fig. S4 (**A**) A bright-field TEM images of the cellular structure with the c-axis out of the viewing plane showing the matrix phase (Sm_2Co_{17} phase) and the cell boundary phase ($SmCo_5$ phase). (**B**) A bright-field TEM image and STEM-EDS mapping with c-axis in the viewing-plane, showing the Sm_2Co_{17} phases (blue), $SmCo_5$ phase (red) and Z-phase (green) and (**C**) the corresponding SAED pattern. (**D**) Energy-dispersive quantitative analysis revealed that the platelets are enriched with Zr atoms (chemical compositions: $Sm_{0.05}Co_{0.67}Cu_{0.04}Zr_{0.23}$), and the Sm_2Co_{17} (chemical

composition: $Sm_{0.10}Co_{0.71}Fe_{0.14}Cu_{0.05}$) and $SmCo_5$ ($Sm_{0.13}Co_{0.54}Cu_{0.26}Fe_{0.07}$) phases are enriched with Cu and Fe, respectively.

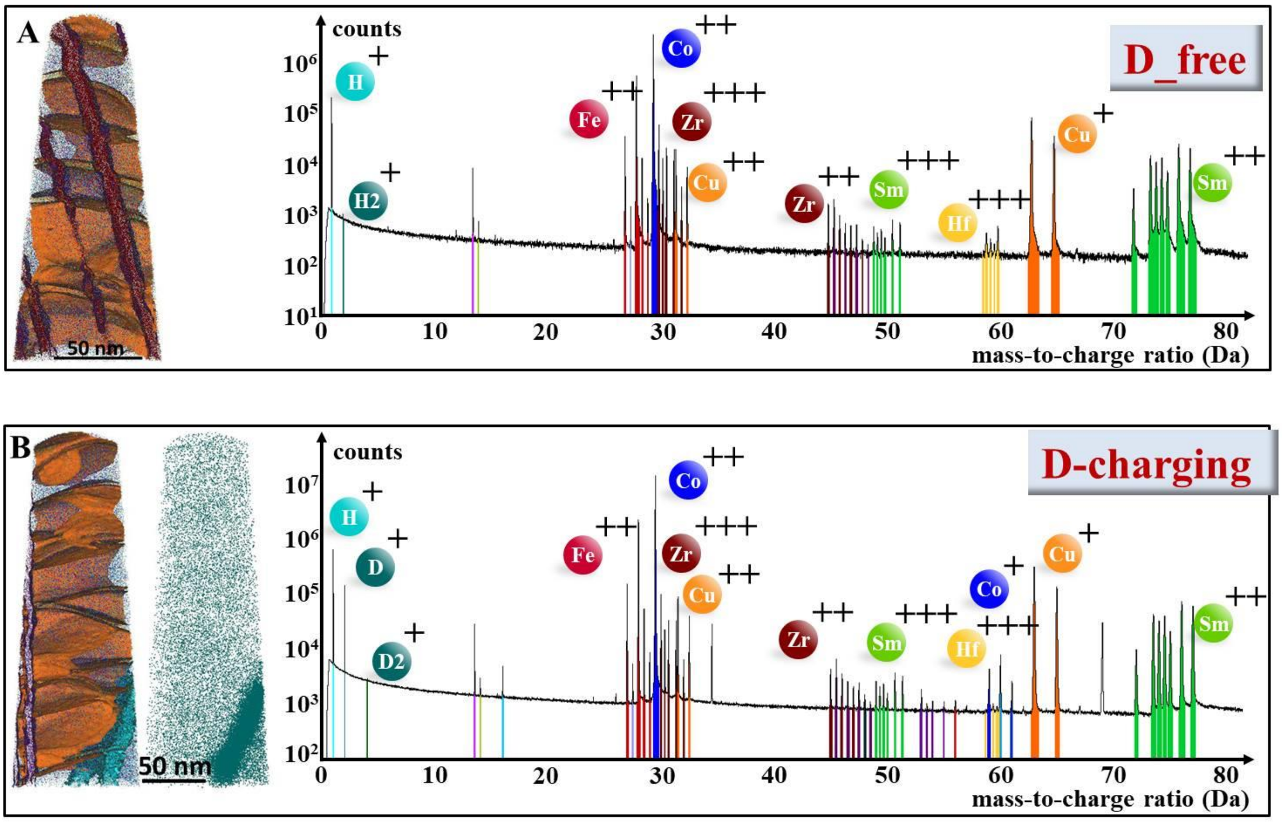

Fig. S5 The mass-to-charge spectrums for (**A**) the as-prepared specimens and (**B**) the deuterium-charged $SmCo_{7.35}$ samples showing that the intensity of the deuterium peaks D^+ at 2Da increased significantly and became

comparable to that of H^+ after the charging. Further, the appearance of D_2^+ peak at 4Da in the deuterium-charged sample confirmed the deuterium charging. In addition, various types of D and H ions, including ZrD^{2+}, HD^+, D^{2+}, CoD^+, $CoHD^+$, CuD^+, $CuHD^+$, FeD+, ZrH^{2+}, CoH^+, FeH^+, CuH^+, etc., were observed in deuterium-charged samples.

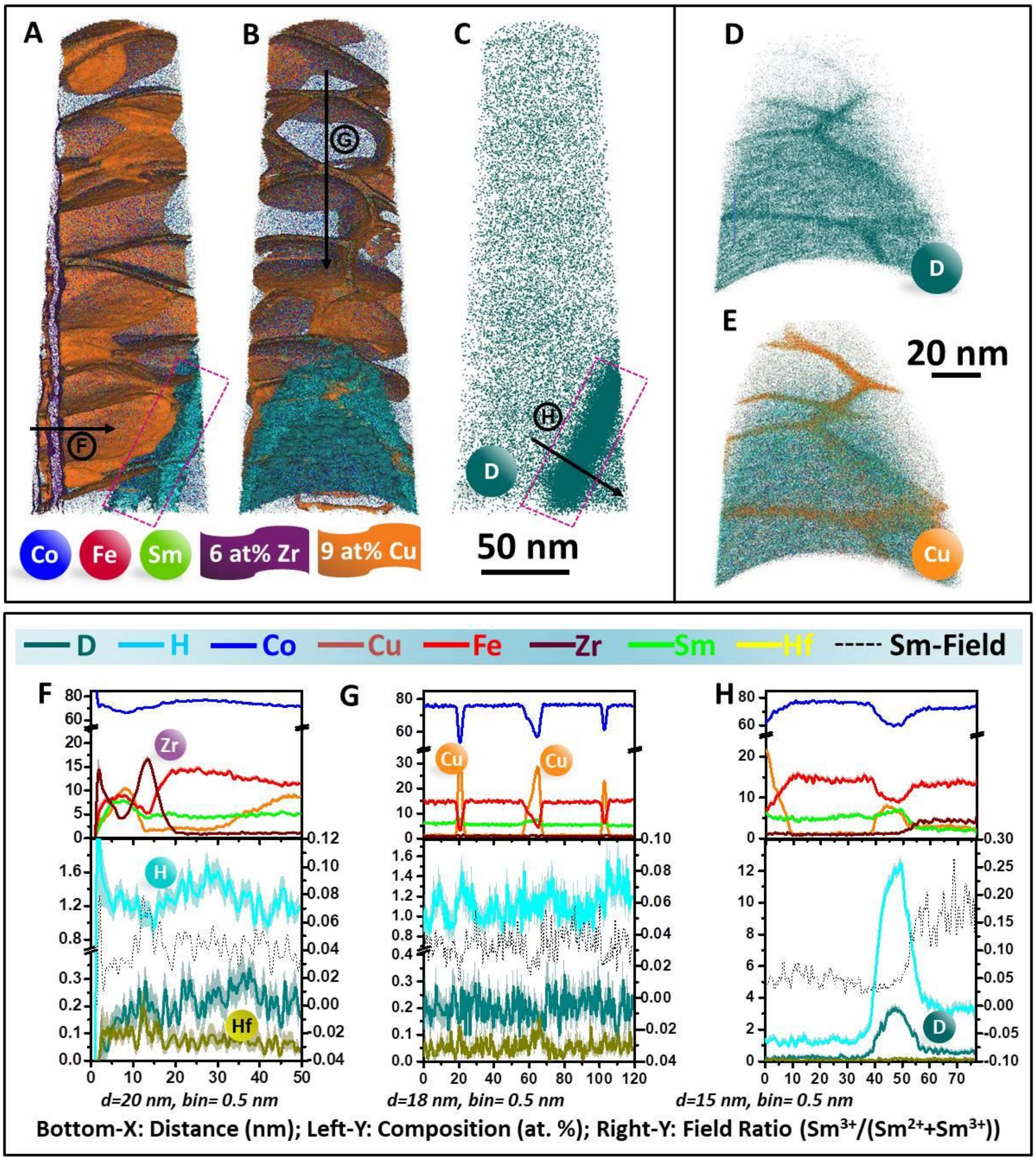

Fig. S6 The 1D Composition profiles through (**F**) the Zr-rich Z-phase (arrowed F in (**A**)), (**G**) the Cu-rich $SmCo_5$ cell boundary (arrowed G in(**B**)), and (**H**) the HAGB (arrowed H in (**C**)). For each case, we evaluated the strength of the electric field by calculating the ratio of $Sm^{3+}/(Sm^{2+}+ Sm^{3+})$, which showed no significant drop at the locations where D and H were detected. This confirms that the observed local segregation of D/H is not related to residual gas ionisation and that the overlap of H2 with D should be minimal.

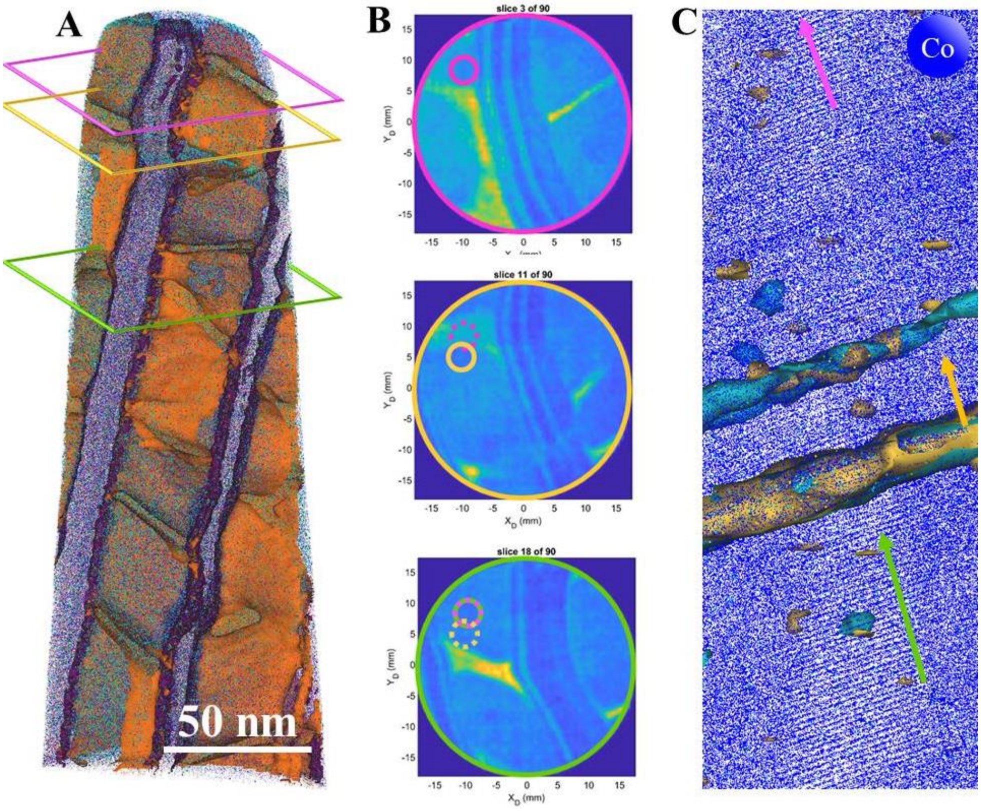

Fig. S7. Pole analysis showing low-angle grain boundary across the the deuterium-segregation layer. (A) The three slices cut from the areas above, across and below the deuterium-segregation layer. (B) The corresponding patterns of the three slices exhibiting the shift of the major pole position. The major pole position of the top slice marked by pink circle shifted to a position marked by a yellow circle in the middle slice, and returned to the

original position as marked by the green circle in the bottom slice. (C) A zoom-in view of the reconstruction of the deuterium-segregation region with 3 at.%H and 0.5at.%Hf iso-concentration surfaces. Note that the lattice planes were interrupted at the grain boundary.

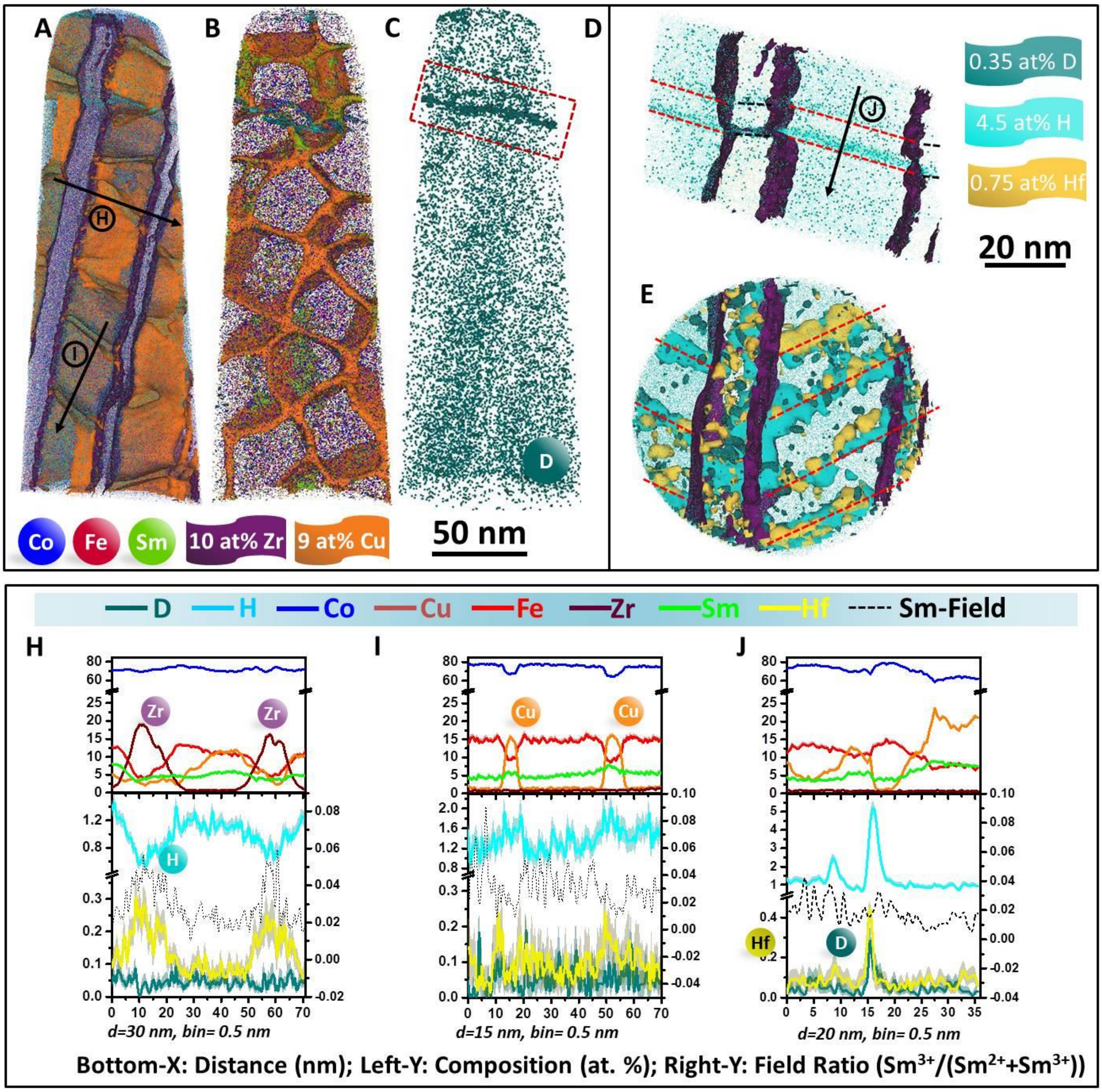

Fig. S8 The 1D Composition profiles through (**H**) Z-phase (arrowed H in (**A**)), (**I**) the $SmCo_5$ cell boundary phase (arrowed I in(**B**)), (**J**) the LAGB (arrowed J in (**C**)). The local variations of electrostatic field (black dash line) obtained by calculating the ratio of $Sm^{3+}/(Sm^{2+}+Sm^{3+})$, was included in these profiles.

	Fe	Co	Ni	Cu	Zr	Sm
Compositi on (at.%)	7.91	55	0.011	6.84	3.25	24.9

Table S1 Chemical composition of the as-received Sm_2Co_{17}-type permanent magnet analyzed by inductively coupled plasma mass spectrometry (ICP-MS).
